# Identification of Early-Stage Breast Cancer with a Minimal Risk of Recurrence by the Breast Cancer Index

**DOI:** 10.1158/1078-0432.CCR-24-3836

**Published:** 2025-03-27

**Authors:** Marie-France Jilderda, Yi Zhang, Valerie Rebattu, Ranelle Salunga, Wilma Mesker, Jenna Wong, Linda de Munck, Tommy Fornander, Bo Nordenskjöld, Olle Stål, Amanda K.L. Anderson, Esther Bastiaannet, Kai Treuner, Gerrit-Jan Liefers

**Affiliations:** 1 Department of Surgery, Leiden University Medical Center, Leiden, the Netherlands.; 2 Biotheranostics Inc., A Hologic Company, San Diego, California.; 3 Department of Research and Development, Netherlands Comprehensive Cancer Organisation, Utrecht, the Netherlands.; 4 Karolinska Institute, Stockholm, Sweden.; 5 Linköping University, Linköping, Sweden.; 6 Epidemiology, Biostatistics and Prevention Institute, University of Zürich, Zurich, Switzerland.

## Abstract

**Purpose:**

This study assessed the prognostic ability of the breast cancer index (BCI) to identify patients at a minimal risk (<5%) of 10-year distant recurrence (DR) who are unlikely to benefit from adjuvant endocrine therapy.

**Experimental Design:**

This prospective translational study included postmenopausal patients with early-stage, hormone receptor–positive N0 breast cancer from the Stockholm (STO-3) trial who underwent surgery alone (“untreated”) or surgery plus adjuvant tamoxifen (“treated”) and from the Netherlands Cancer Registry (surgery alone). The primary endpoint was time to DR. An adjusted BCI model with an additional cutpoint was developed, which stratified patients into four prognostic risk groups.

**Results:**

Across cohorts, 16% to 22% of patients were classified as minimal risk of 10-year DR. In the Stockholm untreated cohort (*n* = 283), risks in the minimal-, low-, intermediate-, and high-risk groups were 2.3%, 15.5% [hazard ratio, 4.71 (95% confidence interval, 1.09–20.29) vs. minimal risk], 19.8% [6.97 (1.61–30.18)], and 35.9% [13.21 (3.07–56.76)], respectively (*P* < 0.001). In the Stockholm treated cohort (*n* = 317), risks were 4.3%, 5.0% [1.16 (0.35–3.85)], 11.7% [2.45 (0.74–8.14)], and 21.1% [5.27 (1.72–16.16); *P* < 0.001]. In the Netherlands Cancer Registry cohort (*n* = 1245), risks were 4.5%, 7.5% [subdistribution hazard ratio, 1.67 (95% confidence interval, 0.81–3.45)], 10.3% [2.40 (1.14–5.03)], and 13.1% [3.13 (1.50–6.55); *P* = 0.005]. BCI risk scores provided additional independent information over standard prognostic factors (likelihood ratio, *χ*^2^ = 7.98; *P* = 0.004).

**Conclusions:**

The adjusted BCI model identified women with early-stage, hormone receptor–positive N0 breast cancer at a minimal risk of DR who may consider de-escalating adjuvant endocrine therapy.

**Translational Relevance**

Many patients with early-stage, hormone receptor–positive breast cancer discontinue adjuvant endocrine therapy before completing the recommended ≥5 years of treatment. Genomic classifiers may guide personalized endocrine therapy to avoid under- and overtreatment. In this prospective translational study, an adjusted breast cancer index (BCI) model with an additional cutpoint identified approximately 20% of patients with invasive, early-stage, hormone receptor–positive N0 breast cancer who had a minimal risk (<5%) of 10-year distant recurrence. This cutpoint was identified using patients in the Stockholm STO-3 trial and validated using patients from the Netherlands Cancer Registry. BCI risk scores provided additional prognostic information beyond standard prognostic factors. The adjusted BCI model may be helpful in identifying patients least likely to benefit from adjuvant endocrine therapy, who might consider shorter treatment durations.

## Introduction

Up to 44% of postmenopausal women with early-stage, hormone receptor–positive (HR+) breast cancer experience disease recurrence within 10 years of diagnosis (1). For over 25 years, adjuvant endocrine therapy (ET) has been the standard of care for reducing the risk of recurrence in this population (2, 3). An Early Breast Cancer Trialists' Collaborative Group (EBCTCG) meta-analysis from 1998 comparing adjuvant tamoxifen versus no tamoxifen in patients with estrogen receptor–positive (ER+) or ER-unknown disease identified reductions in the recurrence rate and improvements in survival for patients who received 5 years of tamoxifen versus 1 or 2 years (2). The adjuvant use of aromatase inhibitors was subsequently supported by positive results from the ATAC and BIG 1-98 trials, which demonstrated reduced recurrence and improved disease-free survival relative to tamoxifen (4, 5).

However, adjuvant ET may not improve outcomes for all patients (1, 6). Moreover, treatment is accompanied by an increased risk of bone toxicities and cardiovascular events with aromatase inhibitors and an increased risk of uterine cancer and deep venous thrombosis with tamoxifen. Poor tolerability is commonly observed with both tamoxifen and aromatase inhibitors (2, 4, 5, 7). A study by Hershman and colleagues (8) found that approximately 30% of patients who initiated ET discontinued treatment prematurely and that another 30% of patients who continued treatment were not fully adherent. A similar study, specifically in older adults, demonstrated that >36% of patients discontinued treatment early, 60% of which was due to toxicity (9). Validated biomarkers are needed to guide clinical decision-making and enable personalized treatment by identifying patients with a very low risk of recurrence who could be safely spared unnecessary adverse effects of adjuvant ET.

The breast cancer index (BCI) is a gene expression–based signature that reports both predictive (10–13) and prognostic results (12–15) to aid in clinical decision-making for extended adjuvant (after 5 years) ET in patients with early-stage, HR+ breast cancer. The predictive component of BCI consists of the HOXB13/IL17BR ratio, an endocrine response biomarker that predicts the likelihood of benefit from extended ET (10, 12, 15, 16). The BCI prognostic score reports the individualized risk of overall (0–10 years after diagnosis) and late (>5 years after diagnosis) distant recurrences (DR) and is calculated using an algorithm that combines the BCI (HOXB13/IL17BR ratio) and the molecular grade index, which interrogates proliferation pathways of five cell-cycle genes (13, 15, 17). The BCI is recommended in the National Comprehensive Cancer Network and American Society of Clinical Oncology clinical practice guidelines for use in clinical decision-making with regard to extended ET (3, 18).

The BCI was previously shown to identify patients with favorable breast cancer–specific survival (BCSS; ref. 19). The current study analyzed the untreated arm of the prospective, randomized Stockholm (STO-3) study to determine an additional BCI cutpoint to identify a group with a minimal risk of 10-year DR even without receiving any adjuvant systemic therapy (15). This cutpoint was prospectively validated in treated patients from STO-3 and in a cohort of patients from the Netherlands Cancer Registry (NCR) who did not receive any adjuvant ET.

## Materials and Methods

### Study design and patient selection

The primary study objective was to examine whether the BCI risk groups were significantly prognostic of DR for women with HR+ N0 breast cancer who did not receive any adjuvant ET. Secondary objectives were to evaluate the prognostic performance of the BCI in patient subgroups according to HER2 status, tumor size, and tumor grade. The study endpoint was time to DR, defined as the time from randomization (Stockholm trial) or date of diagnosis (NCR) to first recurrence at distant sites. Contralateral disease, locoregional recurrence, and other secondary primary cancers were not counted as events and censored.

This analysis included patients from the STO-3 study and a cohort from the NCR.

The prospective, randomized controlled Stockholm trial enrolled patients from 1976 to 1990 and compared 2 or 5 years of adjuvant tamoxifen versus surgery alone in postmenopausal women with invasive, early-stage breast cancer, including 1,780 node-negative (N0) patients (20). The BCI translational study evaluated 283 patients with ER+ disease from the Stockholm cohort who received surgery alone (untreated arm) and 317 patients with ER+ disease who received tamoxifen (treated arm; ref. 15). The cutoff level for ER positivity was set to 1% as previously described (15).

The NCR cohort included patients, ages ≥70 years, with invasive, ER+ N0 breast cancer diagnosed in 2003 to 2009 who had undergone surgery and had not received adjuvant ET or chemotherapy from 45 hospitals throughout the Netherlands (21). Specially trained data managers from NCR collected clinical data on diagnosis, staging, and treatment directly from medical records using international coding rules. The ER status was considered positive if ≥10% of tumor cells showed positive nuclear staining, in compliance with the Dutch national breast cancer guidelines (22). Additional information on comorbidity at the time of diagnosis and recurrences was retrospectively collected from the medical records. Palga, the nationwide network and registry of histo- and cytopathology in the Netherlands, facilitated the registration and distribution of corresponding formalin-fixed, paraffin-embedded tumor blocks acquired during routine treatment.

The current BCI prognostic model stratifies patients into low-, intermediate-, and high-risk groups using validated cutpoints of 5.1 and 6.5 (15). For the current study, untreated patients from the Stockholm cohort were used to determine an additional BCI cutpoint that would identify a subset of low-risk patients with less than 5% risk of 10-year DR even without receiving adjuvant ET. This cutpoint, along with the current cutpoints, was used to stratify patients into four prognostic risk (minimal-, low-, intermediate-, and high-risk) groups. This adjusted BCI model was then validated in the treated arm of the Stockholm cohort to assess the benefit of ET among BCI risk groups. Finally, the adjusted BCI model was validated in the untreated NCR cohort.

Collection and analysis of tumor samples from the Stockholm trial and NCR cohort were approved by institutional review boards. In accordance with the approvals, informed consent from patients was not required. The Stockholm trial was performed in compliance with the Declaration of Helsinki, and the NCR study was done in accordance with the International Council for Harmonisation of Technical Requirements for Pharmaceuticals for Human Use (ICH) Good Clinical Practice guidelines E6(R2).

### BCI molecular testing

BCI analysis using the Stockholm cohort was described previously (15). For the NCR cohort, analysis of BCI gene expression was performed using RT-qPCR with RNA extracted from primary tumor specimens. Tumor sections were prepared by Leiden University Medical Center and shipped to Biotheranostics, Inc. BCI testing was performed in a Clinical Laboratory Improvement Amendments–certified, College of American Pathologists (CAP)–accredited laboratory (Biotheranostics, Inc., a Hologic company), blinded to clinical outcomes, in accordance with Good Clinical Practice. Total extracted RNA was reverse-transcribed. The resulting cDNA was preamplified by PCR using the PreAmp Master Mix Kit (Thermo Fisher Scientific) and analyzed using TaqMan RT-PCR. Samples that passed predetermined quality-control assessments (mean cycle threshold of four reference genes <26.5) were included in final analyses (15). BCI scores were calculated, and predefined cutpoints, including the new cutpoint identified in this study, were used to stratify patients into four risk groups: minimal, low, intermediate, and high risk (15).

### Statistical considerations

Assuming that 20% of patients will be classified as minimal risk with <5% 10-year risk of DR, a minimum of 1,220 patients will be required to ensure that the upper bound of 95% confidence interval (CI) of the risk of DR is not more than 7.5%.

Kaplan–Meier survival analysis was used in the Stockholm study to graphically present the survival curves of the adjusted BCI risk groups; the equality of the survival curves was evaluated using a log-rank test. Hazard ratios (HR) and 95% CI were calculated using Cox proportional hazards models. For the NCR cohort, due to a substantial rate of death of 81% of subjects after a 10-year follow-up, cumulative incidence analysis and subdistribution HR (sHR) from Fine–Gray models with likelihood ratio tests were used, both analyses considering death as a competing risk event. Univariate and multivariate Fine–Gray models with likelihood ratio tests were used to evaluate whether the BCI as a continuous risk index provided additional prognostic information independent of clinical and pathologic factors (age, tumor size, tumor grade, and HER2 status). The BCI was also analyzed in 5-unit increments to dichotomize the BCI and facilitate the comparison of HR with clinical covariates. All analyses were performed with follow-up truncated at 10 years. A two-sided *P* ≤ 0.05 was considered statistically significant. Statistical analyses were done using R (version 4.3.1).

### Data availability

The data analyzed in the current study are not publicly available because they contain patient data and proprietary information. Qualified researchers may request access to de-identified individual participant data by submitting a proposal to the corresponding author, which will be reviewed for scientific merit and feasibility.

## Results

### Patient characteristics

**Figure 1.**
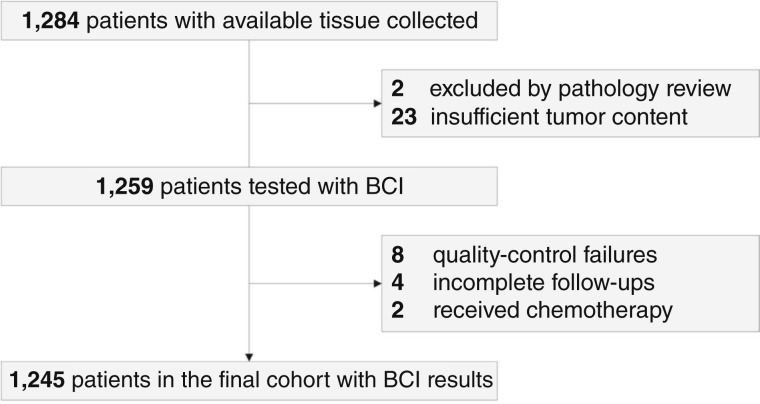
CONSORT diagram for patient selection in the NCR cohort.

Tumor specimens were collected from 1,284 patients from the NCR (Fig. 1). After excluding 39 specimens that had insufficient tumor content or did not meet eligibility criteria, the final dataset included 1,245 patients who did not receive any adjuvant ET.

Clinicopathologic characteristics of the Stockholm and NCR cohorts are shown in Table 1. Most patients in the Stockholm cohort were <70 years of age (untreated, 96.8%; treated, 96.2%); all patients in the NCR cohort were ≥70 years of age. A higher proportion of patients in the Stockholm cohort had T1 tumors (untreated, 81.3%; treated, 81.7%) compared with the NCR cohort (55.0%). The median follow-ups of Stockholm and NCR cohorts were 17 and 12.6 years, respectively.

**Table 1.** Patient clinicopathologic characteristics in the Stockholm and NCR cohorts.

Characteristic	*n* (%)		
	Stockholm untreated (*n* = 283)	Stockholm treated (*n* = 317)	NCR (*n* = 1,245)
Age at surgery, years			
<50	4 (1.4)	2 (0.6)	0
50–59	92 (32.5)	89 (28.1)	0
60–69	178 (62.9)	214 (67.5)	0
70–74	9 (3.2)	12 (3.8)	528 (42.4)
75–79	0	0	256 (20.6)
≥80	0	0	461 (37.0)
Tumor size, mm	0	0	
≤20	230 (81.3)	259 (81.7)	685 (55.0)
>20	51 (18.0)	55 (17.4)	560 (45.0)
Unknown	2 (0.7)	3 (0.9)	0
Tumor grade			
Well	69 (24.4)	67 (21.1)	277 (22.2)
Moderate	175 (61.8)	211 (66.6)	756 (60.7)
Poor	39 (13.8)	39 (12.3)	212 (17.0)
PR status			
PR−	73 (25.8)	72 (22.7)	225 (18.1)
PR+	182 (64.3)	220 (69.4)	962 (77.3)
Unknown	28 (9.9)	25 (7.9)	58 (4.6)
HER2 status			
HER2−	257 (90.8)	295 (93.1)	1,171 (94.1)
HER2+	26 (9.2)	22 (6.9)	74 (5.9)
Adjuvant chemotherapy			
No	283 (100)	317 (100)	1,245 (100)
Yes	0	0	0
Surgery type			
Mastectomy	NA	NA	690 (55.4)
Lumpectomy	NA	NA	555 (44.6)
Locoregional recurrence	31 (11.0)	25 (7.9)	54 (4.3)
DR	56 (19.8)	33 (10.4)	107 (8.6)

Abbreviations: NA, not available; PR, progesterone receptor.

### Identification of the BCI minimal risk cutpoint

Untreated patients from the Stockholm cohort (*n* = 283) were used to refine prognostic risk stratification to identify a subset of low-risk patients with minimal risk of DR even in the absence of any adjuvant ET. A new BCI cutpoint of 3.0 was chosen to ensure a 10-year DR risk of less than 5%; patients below this cutpoint constituted the minimal-risk group.

**Figure 2.**
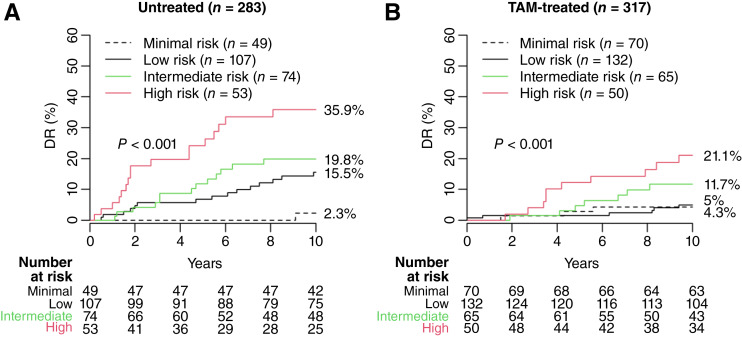
Performance of the adjusted BCI model in the Stockholm cohorts. The new cutpoint for the BCI minimal-risk group was trained using the untreated patients. (**A**) Untreated and (**B**) TAM-treated. TAM, tamoxifen.

In the Stockholm untreated cohort (*n* = 283), 49 (17.3%), 107 (37.8%), 74 (26.1%), and 53 (18.7%) patients were classified as minimal-, low-, intermediate-, and high-risk groups, respectively. The corresponding 10-year risks of DR were 2.3% (95% CI, 0.0–6.6), 15.5% (95% CI, 7.9–22.5), 19.8% (95% CI, 9.5–28.9), and 35.9% (95% CI, 20.4–48.4), respectively (Fig. 2A; Table 2). The adjusted BCI model was significantly prognostic for DR (*P* < 0.001), with HR of 4.71 (95% CI, 1.09–20.29) for low risk, 6.97 (95% CI, 1.61–30.18) for intermediate risk, and 13.21 (95% CI, 3.07–56.76) for high risk versus minimal risk (Fig. 2A; Table 2).

**Table 2.** Prognostic performance of the four BCI risk groups in Stockholm and NCR cohorts.

Cohort	Patients, *n* (%)	DR risk, % (95% CI)	(s)HR^a^, % (95% CI)	*P*
Stockholm untreated	*n* = 283			
BCI minimal risk	49 (17.3)	2.3 (0.0–6.6)	—	<0.001
BCI low risk	107 (37.8)	15.5 (7.9–22.5)	4.71 (1.09–20.29)	
BCI intermediate risk	74 (26.1)	19.8 (9.5–28.9)	6.97 (1.61–30.18)	
BCI high risk	53 (18.7)	35.9 (20.4–48.4)	13.21 (3.07–56.76)	
Stockholm treated	*n* = 317			
BCI minimal risk	70 (22.1)	4.3 (0.0–9.0)	—	<0.001
BCI low risk	132 (41.6)	5.0 (1.0–8.9)	1.16 (0.35–3.85)	
BCI intermediate risk	65 (20.5)	11.7 (3.1–19.5)	2.45 (0.74–8.14)	
BCI high risk	50 (15.8)	21.1 (8.5–32.0)	5.27 (1.72–16.16)	
NCR	*n* = 1,245			
BCI minimal risk	204 (16.4)	4.5 (2.2–8.0)	—	0.005
BCI low risk	508 (40.8)	7.5 (5.4–10.0)	1.67 (0.81–3.45)	
BCI intermediate risk	295 (23.7)	10.3 (7.2–14.2)	2.40 (1.14–5.03)	
BCI high risk	238 (19.1)	13.1 (9.2–17.8)	3.13 (1.50–6.55)	

a HR was used for the Stockholm cohorts; sHR considering death as a competing risk was used for the NCR cohort.

### Validation of the BCI minimal risk cutpoint

Treated patients from the Stockholm cohort (*n* = 317) were used to assess the benefit of ET among BCI risk groups. BCI categorized 70 (22.1%), 132 (41.6%), 65 (20.5%), and 50 (15.8%) patients as minimal-, low-, intermediate-, and high-risk groups with 10-year risks of DR of 4.3% (95% CI, 0.0–9.0), 5.0% (95% CI, 1.0–8.9), 11.7% (95% CI, 3.1–19.5), and 21.1% (95% CI, 8.5–32.0), respectively (Fig. 2B; Table 2). HR were 1.16 (95% CI, 0.35–3.85) for low risk, 2.45 (95% CI, 0.74–8.14) for intermediate risk, and 5.27 (95% CI, 1.72–16.16) for high risk (*P* < 0.001) versus minimal risk (Fig. 2B; Table 2).

Across Stockholm cohorts, patients categorized as low, intermediate, and high risk derived an absolute benefit with tamoxifen, with reductions in risks of DR of 10.5%, 8.1%, and 14.8%, respectively. In contrast, patients categorized as minimal risk did not derive any benefit from adjuvant tamoxifen (−2.0%).

EBCTCG analyses have shown long-term continued recurrence risk well beyond 10 years in patients with HR+ HER2− breast cancer (23). The long follow-up in the Stockholm cohort (median follow-up, 17 years) also allowed the estimation of the 15-year risk of DR. As expected, the 15-year risk for the minimal risk group was higher for both untreated and treated patients at 4.7% (95% CI, 0.0–10.8) and 5.8% (95% CI, 0.1–11.2), respectively, versus the 10-year risk.

### Independent validation of the BCI minimal risk cutpoint

**Figure 3.**
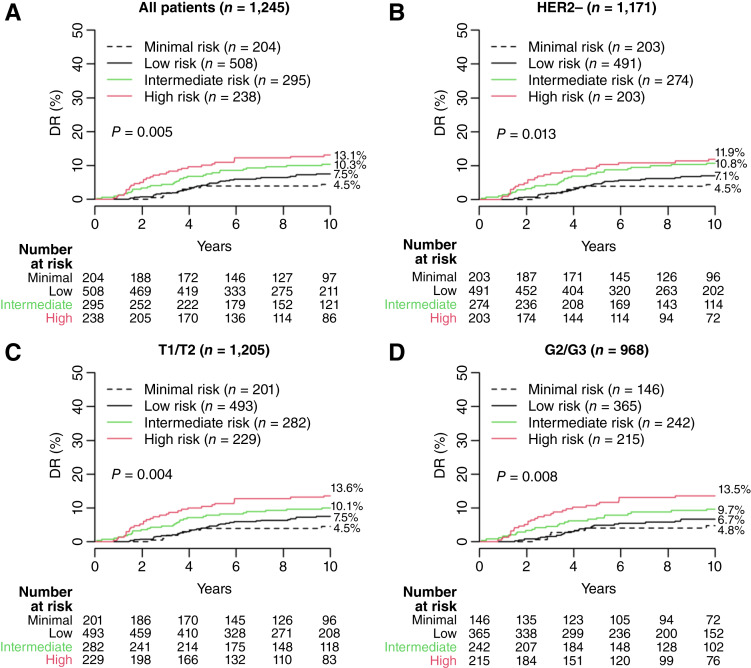
Performance of the adjusted BCI model in the NCR cohort (**A**) overall and (**B–D**) in clinical subsets. G2/G3, grade 2/3; T1/T2, T stage 1/2.

The performance of the adjusted BCI model was externally validated in patients from the NCR cohort. No patient in this cohort received adjuvant ET or adjuvant chemotherapy. In the overall cohort (*n* = 1245), 204 (16.4%), 508 (40.8%), 295 (23.7%), and 238 (19.1%) patients were categorized as minimal-, low-, intermediate-, and high-risk groups, respectively (Fig. 3A; Table 2). The corresponding 10-year risks of DR were 4.5% (95% CI, 2.2–8.0), 7.5% (95% CI, 5.4–10.0), 10.3% (95% CI, 7.2–14.2), and 13.1% (95% CI, 9.2–17.8), respectively (Fig. 3A; Table 2). The sHR were 1.67 (95% CI, 0.81–3.45) for low risk, 2.40 (95% CI, 1.14–5.03) for intermediate risk, and 3.13 (95% CI, 1.50–6.55) for high risk versus minimal risk (*P* = 0.005; Fig. 3A; Table 2).

The prognostic performance of the adjusted model was further evaluated in relevant clinical subgroups. Among patients with HER2− disease (*n* = 1171), 10-year risks of DR were 4.5% (95% CI, 2.2–8.0) for minimal risk, 7.1% (95% CI, 5.0–9.6) for low risk, 10.8% (95% CI, 7.4–14.8) for intermediate risk, and 11.9% (95% CI, 7.9–16.8) for high risk (Fig. 3B). The sHR were 1.58 (95% CI, 0.76–3.28) for low risk, 2.48 (95% CI, 1.18–5.21) for intermediate risk, and 2.93 (95% CI, 1.37–6.25) for high risk (*P* = 0.013), with death as a competing risk event.

Among patients with T1 to T2 disease (*n* = 1205), the 10-year risks of DR were 4.5% (95% CI, 2.2–8.1) for minimal risk, 7.5% (95% CI, 5.4–10.1) for low risk, 10.1% (95% CI, 6.9–14.0) for intermediate risk, and 13.6% (95% CI, 9.5–18.4) for high risk, with death as a competing risk event (Fig. 3C). The sHR were 1.65 (95% CI, 0.80–3.41) for low risk, 2.31 (95% CI, 1.09–4.87) for intermediate risk, and 3.22 (95% CI, 1.54–6.73) for high risk versus minimal risk (*P* = 0.004).

Among patients with grade 2/3 disease (*n* = 968), the 10-year risks of DR were 4.8% (95% CI, 2.1–9.2) for minimal risk, 6.7% (95% CI, 4.4–9.6) for low risk, 9.7% (95% CI, 6.3–13.9) for intermediate risk, and 13.5% (95% CI, 9.4–18.5) for high risk (Fig. 3D). The sHR were 1.39 (95% CI, 0.60–3.20) for low risk, 2.05 (95% CI, 0.89–4.76) for intermediate risk, and 3.10 (95% CI, 1.37–7.01) for high risk versus minimal risk (*P* = 0.008).

To investigate whether the BCI provided independent prognostic information over traditional clinicopathologic factors, univariate and multivariate Fine–Gray models were used. Both tumor size and BCI risk scores were significantly prognostic for 10-year DR in both univariate and multivariate analyses (Table 3). Thus, BCI risk scores provided additional independent prognostic information over standard prognostic factors, with an sHR of 2.19 (1.28–3.74; likelihood ratio *χ*^2^ = 7.98; *P* = 0.004).

**Table 3.** Univariate and multivariate Fine–Gray regression analyses of BCI in the NCR cohort.

Variable	Univariate analysis		Multivariate analysis	
	sHR (95% CI)	*P*	sHR (95% CI)	*P*
Age^a^	1.11 (0.85–1.44)	0.45	0.88 (0.65–1.19)	0.41
Tumor size (>20 mm vs. ≤20 mm)	1.95 (1.33–2.87)	0.001	2.12 (1.35–3.34)	0.001
Tumor grade (G2/G3 vs. G1)	1.00 (0.64–1.56)	0.99	1.08 (0.67–1.75)	0.75
HER2 (HER2+ vs. HER2−)^b^	1.87 (1.01–3.45)	0.05	1.72 (0.91–3.26)	0.10
BCI^c^	2.49 (1.50–4.14)	<0.001	2.19 (1.28–3.74)	0.004

Abbreviation: G, grade.

a Age was analyzed in 10-year increments; all patients were at least 70 years of age.

b 5.9% of patients were HER2+.

c BCI was analyzed as a continuous variable with 5-unit increments.

## Discussion

For over 25 years, the standard-of-care treatment for HR+ breast cancer has included at least 5 years of adjuvant ET. This standard was established before the emergence of genomic classifiers, which have subsequently been shown to provide important information on the tumor biology to improve selection of patients for chemotherapy and ET. Both therapies can be poorly tolerated and carry risks of toxicities and serious adverse effects, which in the case of ET can lead patients to discontinue treatment early before completing the recommended 5 years. Nonadherence to ET is well documented. One landmark study found that fewer than half of patients with breast cancer took adjuvant ET for the full duration at the optimal schedule (8). Furthermore, a new EBCTCG meta-analysis revealed that the risk of DR in patients with ER+ breast cancer from 2000 to 2009 was reduced by 25% compared with that from 1990 to 1999 (24). The goal of the current study was to determine whether a genomic classifier could optimize patient selection for adjuvant ET and identify patients at minimal risk of DR who may consider shorter durations of ET.

The BCI identified approximately 20% of patients at a minimal (<5%) 10-year risk of DR, even without having received any adjuvant ET. When evaluated in the Stockholm cohort, all patients categorized as BCI low, intermediate, and high risk derived substantial benefit (>8% in reduction of 10-year risk of DR) from adjuvant tamoxifen. In contrast, patients categorized as BCI minimal risk derived no benefit from tamoxifen, with 10-year risks of DR of 2.3% and 4.3% in the untreated and treated cohorts, respectively. The numerically lower risk of DR observed in the untreated cohort may be attributed partly to the BCI model being trained in these patients and partly due to the low event rate. An interesting observation in treated patients from the Stockholm cohort was that minimal- and low-risk patients had similar risk profiles because of the difference in ET benefit experienced by low-risk, but not by minimal-risk, patients. Untreated patients from the NCR provided a rare and unique opportunity to evaluate the prognostic performance of the BCI in a large cohort of older patients with HR+, early-stage breast cancer who did not receive adjuvant ET or chemotherapy. Data from the NCR cohort revealed a 10-year risk of DR of 4.5% for minimal-risk patients, supporting the performance observed in the Stockholm cohort. Furthermore, the numeric risk estimates of DR in high-risk patients in the NCR cohort were substantially lower than those in the Stockholm cohorts, as the NCR cohort was restricted to patients aged over 70 years. This resulted in a significant number of deaths (81%) within the 10-year follow-up and might underestimate the risk of DR, albeit statistical analyses considered death as a competing risk.

An EBCTCG meta-analysis from 20 trials with more than 20,000 patients showed that 5 years of tamoxifen reduced the risk of DR and had a similar effect on the incidence of local recurrence and contralateral breast cancer (1). DR represented 71% of the first recurrence events, whereas local recurrence and contralateral breast cancer represented 13% and 15%, respectively. Given that many local recurrences are eventually followed by a DR and that DR is the clinically most important and frequent recurrence event leading to significant mortality, an endocrine treatment decision focused on DR risk is a sensible strategy to identify patients at a very low risk of recurrence. These findings suggest that the new BCI minimal risk cutpoint may identify women with HR+ N0 breast cancer at a sufficiently low risk of DR who would derive minimal benefit from adjuvant ET and could consider de-escalating treatment.

The 70-gene assay (MammaPrint) is validated for the ability to classify the risk of BCSS and DR in early-stage breast cancer (25–28). An initial validation of the 70-gene assay for ultralow-risk classification was based on BCSS (26, 27). In the Stockholm trial, the assay categorized 14% of untreated patients as ultralow risk, with 94% BCSS at 20 years (27). However, these patients only had 90% BCSS at 20 years when breast cancer deaths after contralateral breast cancer were included. In this context, in a previous BCI study of the Stockholm trial, an adjusted BCI cutpoint of 3.4 (comparable with the cutpoint of 3.0 in this study) classified 21% of untreated patients as minimal risk with 92% BCSS at 20 years (19). In the MINDACT trial, 15% of patients with T1 to T3, N0 to N1, and M0 breast cancer were also identified as ultralow risk with an 8-year BCSS of 99.6% and distant metastasis–free interval of 97.0% (29). Of these, at least 83% received ET. In the FOCUS cohort of 418 HR+ patients, ages ≥70 years, the 70-gene assay categorized 12% of patients as ultralow risk of 10-year DR (28). Among ultralow-risk patients, 2% developed DR within 10 years after diagnosis; however, 52% had been treated with adjuvant ET. Unlike these studies, which either only reported BCSS that ignores DR that had not yet resulted in death within the follow-up period or reported DR but included patients who were treated with ET, this study included all DR as the study endpoint in a large cohort of patients who did not receive any adjuvant systemic treatments and thus may represent the true risk of breast cancer in older patients classified as minimal risk.

The potential for selection bias should be considered a study limitation, as the NCR cohort is derived from a nationwide registry and reasons for withholding ET are not recorded. However, the registry provides a large, heterogeneous patient population reflecting real-world evidence. Second, the NCR cohort only included patients of >70 years of age, and most patients (81%) died within a 10-year follow-up, largely due to other causes. Therefore, death was a significant competing risk for DR, which might underestimate the risk of DR versus the Stockholm cohorts, especially for the high-risk patients, even with the statistical models attempting to account for the competing risk. In addition, due to older patient age and fewer than 10% of patients with HER2+ disease in the NCR cohort, the multivariate analysis might be limited to examine the prognostic ability of age and HER2 status. Additional studies are warranted to evaluate the risk of DR beyond 10 years in patients with BCI minimal risk and to evaluate the minimal risk cutpoint in younger patients.

In conclusion, the results of the current study support BCI as a biomarker to personalize adjuvant endocrine treatment decisions to avoid over- and undertreatment of patients. If these findings are supported by future studies, physicians may consider ordering BCI at diagnosis or within the first 5 years to inform the risk of DR and consider potential de-escalation of adjuvant ET in minimal-risk patients. This information may be particularly helpful for patients who struggle with the adverse effects of ET as well as older patients with comorbidities.
